# EHMTI-0029. Medicated headache mask

**DOI:** 10.1186/1129-2377-15-S1-G15

**Published:** 2014-09-18

**Authors:** M Hyson

**Affiliations:** 1 Neurology, University of Nevada, Las Vegas, USA

## Introduction

A topical treatment for migraine and/or tension headaches.

## Aims

This study was performed to determine the efficacy of an anticephalgic photoprotective mask in conjunction with a topical medication containing bryonia and rhus toxicodendron in the treatment of migraine and/or tension headache.

## Methods

Thirty-three patients were given masks and tubes of topical medication containing the bryonia and rhus toxicodendron. They were instructed to apply the medication to their frontalis and/or temporalis regions in the event they should suffer a headache and apply a photoprotective mask. Furthermore, they were instructed to take their usual oral or parenteral medications if required for the relief of the headache. They subsequently filled out forms rating the degree of relief which they attributed to the topical medication and the mask using a 0-10 scale. At the interview following the completion of their participation in the study, the patients were also simply asked if this form of treatment helped or not.

## Results

Thirty out of 33 patients stated the medication and the mask were effective over and above the normal degree of relief they were receiving from their oral and/or parenteral medications. This study demonstrated a significant efficacy rate (91%) in the treatment of migraine and/or tension headache with the anticephalgic mask in conjunction with a topical cream containing bryonia and rhus toxicodendron.

## Conclusions

This study demonstrated a significant efficacy rate in the treatment of migraine and/or tension headache with the anticephalgic mask in conjunction with a topical cream containing bryonia and rhus toxicodendron.

No conflict of interest.

